# Artificial intelligence-based quantitative analysis of interocular retinal vascular differences in school-age children with mild to moderate anisometropia

**DOI:** 10.3389/fmed.2026.1829476

**Published:** 2026-07-01

**Authors:** Zhixing Xu, Yuke Ji, Yuting Hu, Zhengyang Tao, Zongyue Lv, Guorui Hu, Minmin Li, Xiaoyan Wang, Hongwei Deng, Zefeng Kang

**Affiliations:** 1 The Second Clinical Medical College, Jinan University, Shenzhen, Guangdong, China; 2 Shenzhen Eye Hospital, Jinan University, Shenzhen, Guangdong, China; 3 Department of Ophthalmology and Optometry, Fujian Medical University, Fuzhou, China; 4 Department of Strabismus and Pediatric Ophthalmology, Shenzhen Eye Hospital, Shenzhen Eye Medical Center, Southern Medical University, Shenzhen, Guangdong, China; 5 Eye Hospital, China Academy of Chinese Medical Sciences, Beijing, China

**Keywords:** anisometropia, artificial intelligence, fundus tessellation, interocular difference, peripapillary atrophy, retinal vasculature, school-age children

## Abstract

**Objective:**

To apply artificial intelligence (AI) technology for the quantitative analysis of interocular retinal vascular differences in color fundus photographs (CFP) among school-age children with mild to moderate anisometropia and to investigate their correlation with the degree of anisometropia.

**Methods:**

In this retrospective cross-sectional study, 33 children aged 6–9 years with mild to moderate anisometropia were enrolled. CFPs of both eyes were acquired. A deep learning-based AI algorithm was employed to quantitatively analyze interocular differences in the degree of fundus tessellation (FT), the extent of peripapillary atrophy (PPA), optic disc morphology, and retinal vascular characteristics. The correlations between these differences and the interocular spherical equivalent (SE) difference were assessed.

**Results:**

Eyes with lower SE values exhibited significantly higher FT density, larger FT areas across all regions, greater PPA area and extent, a larger optic disc ovality index, higher total vessel length density, and higher arterial fractal dimensions (D0, D1) compared to eyes with higher SE values. In contrast, the horizontal cup-to-disc ratio and the average venous tortuosity were significantly lower in the lower SE eyes (all *P* < 0.05). Correlation analysis revealed that the interocular differences in the horizontal and vertical diameter ratios of PPA to the optic disc (*r* = 0.433, *P* = 0.012; *r* = 0.368, *P* = 0.035, respectively) and in the arterial singularity length (SL_a) (*r* = 0.397, *P* = 0.022) were positively correlated with the degree of anisometropia. No significant linear associations were found between the degree of anisometropia and the interocular differences in other fundus parameters (all *P* > 0.05).

**Conclusion:**

Significant interocular differences in fundus structures are present in children with mild to moderate anisometropia. The magnitude of interocular difference in the PPA-to-optic disc diameter ratio shows a significant correlation with the degree of anisometropia. AI-based quantitative analysis provides an objective imaging basis for the early identification of anisometropia.

## 1 Introduction

Anisometropia is a clinical condition characterized by an asymmetry in the refractive error between the two eyes (1), It is associated with various visual problems, including strabismus, amblyopia, hyperopia, and myopic progression (2). It is estimated that approximately 10% of adults are affected by anisometropia (3). The prevalence of anisometropia changes dynamically with age, showing a general pattern: it may decrease gradually during early childhood with ocular development, increase during school age due to myopia onset, and stabilize in adulthood. If not corrected promptly, the eye with higher refractive error in anisometropia is prone to developing amblyopia, which can severely impact visual function (4). Anisometropia manifests not only as differences in refractive error and axial length but may also be accompanied by alterations in fundus structures (5). Existing research on anisometropia has primarily focused on comparing interocular differences in refractive error and axial length parameters. Systematic quantitative assessment of interocular differences in fundus morphology remains relatively scarce, especially among school-age children undergoing rapid ocular development (6). Previous studies have found that characteristic morphological changes in the fundus, such as alterations in fundus tessellation (FT), optic disc, and retinal vasculature, often occur during the onset and progression of myopia. These findings provide important clues for understanding the fundus morphological changes associated with anisometropia.

Color fundus photography (CFP) is an ideal method for assessing retinal structure in children due to its non-invasiveness, ease of operation, and intuitive imaging (7–9). Using CFP, ophthalmologists can systematically analyze subtle changes in fundus features associated with variations in refractive status. This can provide a more precise basis for interventions aimed at delaying or controlling the progression of anisometropia. However, in current clinical practice, the identification and quantification of fundus features still heavily rely on physician visual assessment. This process is highly subjective, time-consuming, labor-intensive, and susceptible to inconsistencies. Therefore, developing a system capable of automatic segmentation and quantitative analysis of fundus structures without manual intervention or outlining holds significant clinical need and application value. In recent years, artificial intelligence (AI) image processing techniques have advanced continuously (10, 11), These techniques can now effectively identify subtle textural differences in CFP images that are indistinguishable by the human eye (12). Shao et al. (12) employed AI technology to quantitatively assess FT density (FTD) and found it was significantly correlated with parameters such as thinner choroidal thickness and longer axial length (12, 13). Liu et al. (14) used a deep learning model to analyze CFP images of children with hyperopia. They found that eyes with high hyperopia had larger average vessel diameter and smaller vertical cup-to-disc ratios, further confirming the sensitivity of AI in capturing fundus parameters (14). AI-based quantitative analysis of CFP holds promise for more objectively revealing subtle early fundus changes in anisometropia. If these changes can be identified early, before anisometropia progresses to high myopia, and timely interventions such as refractive correction are implemented, it could significantly help reduce the incidence of amblyopia (15, 16). Therefore, this study aims to apply AI technology to quantitatively analyze CFP images from both eyes of school-age children with early-stage anisometropia. We will systematically compare interocular differences in the degree of FT, optic disc morphology, and retinal vascular characteristics. Furthermore, we will explore the correlation between these differences and the degree of anisometropia. The goal is to preliminarily identify sensitive fundus indicators associated with anisometropia. This will lay the foundation for establishing future early prediction and monitoring models, thereby providing an objective and quantitative imaging basis for the precise prevention and control of anisometropia.

## 2 Materials and methods

### 2.1 Participants

We retrospectively analyzed the clinical data of children aged 6–9 years who visited the Shenzhen Eye Hospital between 2023 and 2025. All participants had undergone at least one cycloplegic refraction examination and one CFP examination during the same visit. Mild to moderate anisometropia was defined as an interocular difference in spherical equivalent (SE) of 1.00 diopters (D) ≤ SE < 3.00 D (17–20). Hyperopic anisometropia was defined as both eyes having SE ≥ 0.00 D. Myopic anisometropia was defined as both eyes having SE < 0.00 D. Mixed anisometropia was defined as one eye having SE ≥ 0.00 D and the fellow eye having SE ≤ 0.00 D (21). Based on the interocular SE difference, eyes were categorized into a higher-SE group and a lower-SE group. Participants meeting any of the following criteria were excluded: (1) corrected visual acuity < 0.7, or a prior diagnosis of amblyopia; (2) high myopia (spherical power ≥ −6.00 D in either eye); (3) the presence of severe inflammation, photophobia, or epiphora in either eye; (4) the presence of organic ocular disease in either eye; (5) a diagnosis of color vision deficiency (color weakness or color blindness); or (6) incomplete data.

### 2.2 Refractive examination

A standard logarithmic E-chart (WB-1112E, Wenbang, China) was placed at a distance of 5 meters. Cycloplegia was induced using compound tropicamide eye drops (Santen, Japan). Three drops were administered at 10-min intervals to ensure adequate pupillary dilation. At least 10 min after the last instillation, cycloplegic refraction was performed. The spherical and cylindrical powers were recorded electronically.

### 2.3 Fundus photography examination

Color fundus images were captured by a single operator in a dark room using a Canon CR-2 PLUS AF fundus camera. For each participant, two 45-degree images centered on the optic disc were acquired for each eye, and the best-quality image from each eye was selected for analysis. Image quality was independently evaluated by two experienced ophthalmologists according to the predefined criteria, and Cohen’s kappa coefficient was calculated to assess inter-rater reliability, yielding κ = 0.89 (substantial agreement, *P* < 0.001). Discrepant assessments were resolved through consensus discussion between the two raters. The fundus photographs met the following criteria: (1) The quality of the color fundus photo was sufficient to identify ≥ 90% of the vessels; (2) The image was centered on the optic disc at a 45-degree angle, with key fundus structures such as the optic disc and macula clearly visible; (3) The imaging area was free of shadows or bright reflections that could interfere with interpretation; (4) The photo was properly exposed, avoiding overexposure or underexposure affecting image quality; (5) The photo contained no blurring factors, such as lens stains, ptosis, long eyelashes, or significant motion artifacts.

### 2.4 Image analysis

**FIGURE 1:**
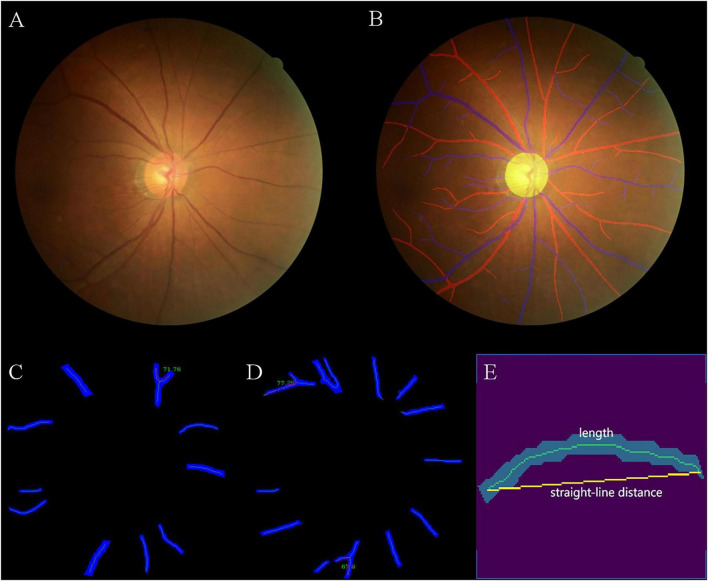
Measurement of vascular parameters. **(A)** Input fundus image. **(B)** Optic disc and artery and vein segmentation, blue for veins, red for arteries, yellow for optic disc. **(C)** Example of the branching angle of artery. **(D)** Example of the branching angle of vein. **(E)** Example of the average curvature.

Color retinal images and their spacing were input to calculate metrics such as Fractal Dimension, branching angle-related parameters, length-related parameters, Central Retinal Arteriolar Equivalent (CRAE), Central Retinal Venular Equivalent (CRVE), Arteriolar-to-Venular Ratio (AVR), etc. The results were saved in a CSV file, with the suffix “_a” for artery-related metrics and “_v” for vein-related metrics (22). We first performed segmentation of the optic disc using HR-Net, which features high-resolution semantic feature representations that are well-suited for accurate segmentation (23, 24). The HR-Net was trained on several public retinal fundus segmentation datasets (DRIVE, HRF, RETA, and Fundus-AVSeg). The model was implemented using PyTorch, with the Adam optimizer, an initial learning rate of 1 × 10^−4^, a batch size of 8, and a total of 100 training epochs. Data augmentation strategies, including random rotation, flipping, scaling, and brightness adjustment, were applied to prevent overfitting. A 5-fold cross-validation strategy was employed on the datasets, and model performance was evaluated using the Dice similarity coefficient (DSC) and accuracy. The final HR-Net achieved a mean DSC of 0.92 ± 0.03 for optic disc segmentation and a mean accuracy of 0.95 ± 0.02 for retinal artery/vein segmentation, demonstrating high accuracy and robustness. After segmentation, we divided the retinal area into Zone A, B, and C according to the distance from the optic disc: Zone A (the region from 0 to 0.5 disc diameter from the edge of the optic disc), Zone B (the region from 0.5 to 1 disc diameter from the edge of the optic disc), and Zone C (the region from 1 to 2 disc diameters from the edge of the optic disc). Subsequently, we segmented retinal arteries and veins again using HR-Net. The resulting masks were skeletonized to extract the centerline of each vessel section. Bifurcation points were obtained by applying a morphological operation on the extracted centerlines. We analyzed four different fractal dimensions of arteries and veins: the Capacity dimension (D0, reflecting the spatial occupancy of the vascular network; higher values indicate a more complex and dense retinal arterial network), Entropy dimension (D1, reflecting the heterogeneity of vascular distribution), Correlation dimension (D2), and Singularity Length (SL, a fractal parameter quantifying the local structural irregularity of vessels; higher SL indicates increased local morphological heterogeneity of arteries), respectively. Larger values in fractal dimension represent a more complex pattern and vice versa. We calculated the average branch angle (angle_avg, the mean angle between parent and daughter vessels at bifurcation points; abnormal branching angles are associated with altered hemodynamics), average branch asymmetry (asymmetry_avg), and average number of branches (branch_avg) for arteries and veins in Zone C, following the definition in Sun et al.’s work (25). If there were no branches in Zone C, the corresponding field in the table was marked as missing data. We further calculated the average length (length_avg), average tortuosity (curvature_avg, defined as the ratio of the actual integral length of the vessel segment centerline to the straight-line distance between its two endpoints, with a smaller value indicating a straighter vessel; abnormally high tortuosity is associated with vascular endothelial dysfunction and blood-retinal barrier damage), overall length density (vessel_length_density, the total length of retinal vessels per unit fundus area, reflecting the density and distribution of the superficial retinal capillary network; lower density indicates potential retinal hypoperfusion), and vessel area density (vessel_density) for arteries and veins in Zone C. We further calculated CRAE, CRVE, and AVR (the ratio of CRAE to CRVE, a classic marker of retinal microvascular status) based on the Knudtson formula (26), as shown below:


$$W_{a\,r\,t\,e\,r\,y}=0.88\,\sqrt{W_{1}^{2}+W_{1}^{2}}$$

$$W_{v\,e\,i\,n}=0.95\,\sqrt{W_{1}^{2}+W_{1}^{2}}$$

where W_1_ and W_2_ represent the narrower and wider retinal branches (Figure 1).

#### 2.4.1 Fundus tessellation and peripapillary atrophy

FT was manually labeled to train a deep learning network. The images in the current study were segmented using this trained network. Manual examination of the segmentation result was performed, and manual correction was applied when necessary to ensure quality. FT area and density were calculated following the definition in a previous study (12). Area (FT) = number of pixels labeled as tessellation * (pixel_spacing_x * pixel_spacing_y).

**FIGURE 2:**
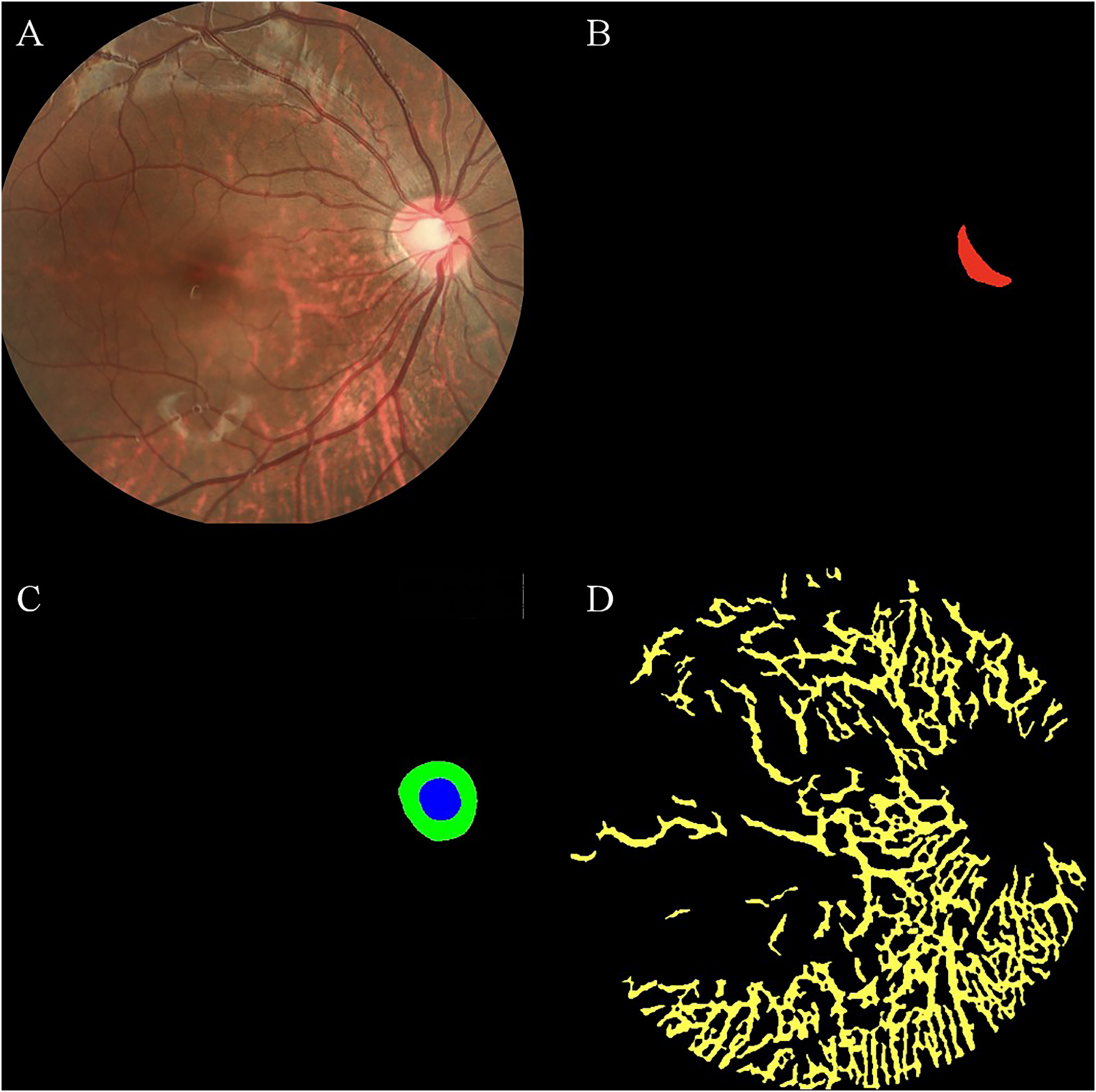
The results of feature recognition. **(A)** The original colour fundus photograph. **(B)** PPA recognition. **(C)** The optic cup recognition is marked in blue and the optic disc recognition is marked in green. **(D)** Blood vessel recognition. PPA, Peripapillary atrophy.

Based on the extracted tessellated area, we calculated the average tessellation area per unit area on the fundus to obtain: Density (FT) = Area (FT)/Total Fundus Area, where the total fundus area was obtained by applying a threshold and a morphological filter on the image to remove the black background. PPA was segmented using HR-Net as described in Wang et al. (23). The maximum vertical distance between two points on the PPA was defined as its height. The maximum thickness of the PPA was defined as its width. The area ratio between the PPA and the optic disc was calculated as a measure of the severity of the PPA (Figure 2).

### 2.5 Statistical analysis

The normality of continuous variables was assessed using the Shapiro-Wilk test. Data following a normal distribution are presented as mean ± standard deviation. Non-normally distributed data are presented as median (first quartile, third quartile). For paired data from the same subject, the interocular difference was tested for normality. When the assumption of normality was satisfied, a one-sample *t*-test was used to estimate the mean and 95% confidence interval; otherwise, the Wilcoxon signed-rank test was employed along with the Hodges-Lehmann estimator to estimate the median and 95% confidence interval. Interocular differences were calculated as the higher-SE eye minus the lower-SE eye. When data were normally distributed, Pearson correlation was used to analyze the association between the interocular SE difference and all fundus parameters; otherwise, Spearman correlation was applied. The false discovery rate (FDR, Benjamini-Hochberg method) was used to adjust *P*-values for multiple outcome comparisons. All analyses were conducted using R software (version 4.2.2; R Foundation for Statistical Computing). A two-sided *P* < 0.05 was considered statistically significant.

## 3 Results

### 3.1 Baseline characteristics

**FIGURE 3:**
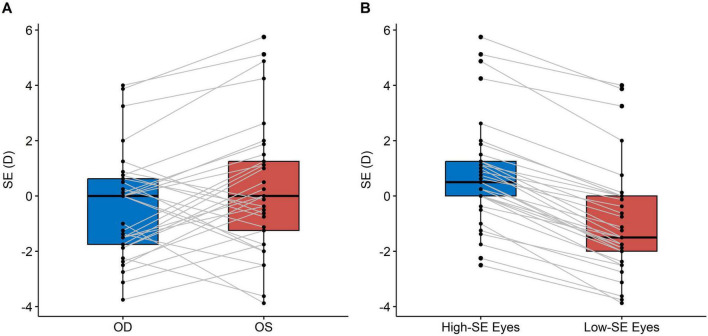
Distribution of spherical equivalent (SE) in patients with myopic anisometropia (*n* = 33). **(A)** Between right and left eyes. **(B)** Between low-SE eye and high-SE eye. D, Diopter; OD, right eyes; OS, left eyes.

This study included 33 participants (66 eyes), among whom 9 (27.27%) had hyperopic anisometropia, 13 (39.39%) had myopic anisometropia, and 11 (33.33%) had mixed anisometropia. There were 15 males (45.45%) and 18 females (54.55%). The mean age of the participants was 7.97 ± 1.13 years. The median spherical equivalent (SE) was 0.00 D [interquartile range (IQR): −1.75, 0.62] in the right eyes and 0.50 D (IQR: −1.25, 1.25) in the left eyes, and the difference between the right eye SE and left eye SE was statistically significant [median difference: −0.56, 95% CI: (−1.62, −0.00), *P* = 0.041]. In the higher-SE eye group, the median SE was 0.50 D (IQR: 0.00, 1.25); in the lower-SE eye group, the median SE was −1.50 D (IQR: −2.00, 0.00). The difference between the higher-SE and lower-SE eyes was statistically significant [median difference: 1.75, 95% CI: (1.50, 2.00), *P* < 0.001] (Table 1 and Figure 3).

**TABLE 1:** Baseline characteristics.

Parameters	High-SE eyes	Low-SE eyes	Difference (95% CI)	*P*-value
SE	0.50 (0.00, 1.25)	−1.50 (−2.00, 0.00)	1.75 (1.50, 2.00)	<0.001
**Spherical power**	0.75 (0.00, 1.50)	−1.25 (−1.75, 0.50)	1.87 (1.62, 2.00)	<0.001
**Cylindrical power** ^†^	−0.97 ± 0.99	−0.88 ± 0.83	−0.13 (−0.50, 0.25)	0.511

SE, Spherical Equivalent.

**^†^***P*-value and Difference (95% CI) were calculated by *t*-test. Others were calculated by Wilcoxon signed rank test and Hodges-Lehmann estimator.

### 3.2 Intergroup comparison between higher-SE and lower-SE Eyes

Regarding the degree of FT, the lower-SE eyes exhibited a significantly higher average FT density (FTD) (*P* = 0.003). The FT area was also significantly larger in the Lower-SE eyes for the global fundus, macular region, and peripapillary region (*P* = 0.003, *P* = 0.002, *P* = 0.008, respectively) (Table 2).

**TABLE 2:** Characteristics of the fundus tessellation in anisometropic patients.

Parameters	Low-SE eyes	High-SE eyes	Difference (95% CI)	*P*-value	Adjusted *P*-value
FTD	0.00 (0.00, 0.03)	0.02 (0.00, 0.06)	−0.02 (−0.04, −0.01)	0.003	0.013
Global tessellation area (mm^2^)	0.00 (0.00, 1.70)	0.90(0.00, 3.70)	−1.30 (−2.10, −0.60)	0.003	0.013
Macular tessellation area (mm^2^)	0.00 (0.00, 0.50)	0.20 (0.00, 1.50)	−0.80 (−1.20, −0.40)	0.002	0.012
Peripapillary tessellation area (mm^2^)	0.00(0.00, 1.30)	0.80(0.00, 3.00)	−0.80 (−1.25, −0.25)	0.008	0.030

FTD, fundus tessellated density; SE, Spherical Equivalent. *P*-values and Differences (95% CI) were calculated by Wilcoxon signed rank test and Hodges-Lehmann estimator. FDR corrections (the Benjamini-Hochberg method) were done for multiple outcomes in anisometropic patients.

In terms of optic disc morphology, the Lower-SE eyes showed a larger area of peripapillary atrophy (PPA) (*P* = 0.002), as well as greater PPA width and height (*P* = 0.027, *P* = 0.016, respectively). Furthermore, both the horizontal and vertical diameter ratios of PPA to the optic disc were significantly higher in the Lower-SE eyes (*P* = 0.003, *P* = 0.014, respectively). The horizontal cup-to-disc ratio was significantly lower in the lower-SE eyes (*P* = 0.031), while no significant difference was found in the vertical cup-to-disc ratio (*P* = 0.315). Both the vertical and horizontal optic disc diameters were significantly smaller in the lower-SE eyes (*P* = 0.010 and *P* < 0.001, respectively). The horizontal cup diameter was also smaller in the lower-SE eyes (*P* < 0.001), while the optic disc ovality index was greater (*P* < 0.001) (Table 3).

**TABLE 3:** Characteristics of the optic disc morphological in anisometropic patients.

Parameters	Low-SE eyes	High-SE eyes	Difference (95% CI)	*P*-value	Adjusted *P*-value
Area ratio of the PPA to the optic disc	0.00 (0.00, 0.03)	0.13 (0.00, 0.27)	−0.13 (−0.24, −0.05)	0.002	0.012
Width of the PPA (μm)	0.00 (0.00, 250.00)	263.00 (0.00, 422.00)	−218.50 (−436.00, −31.50)	0.027	0.065
Height of the PPA (μm)	0.00 (0.00, 413.00)	785.00 (0.00, 1202.00)	−501.00 (−777.00, −82.00)	0.016	0.044
Horizontal diameter ratio of the PPA to the optic disc	0.00 (0.00, 1.01)	1.04 (0.00, 1.12)	−0.56 (−0.94, −0.09)	0.003	0.013
Vertical diameter ratio of the PPA to the optic disc	0.00 (0.00, 1.05)	1.05 (0.00, 1.19)	−0.55 (−1.02, −0.07)	0.014	0.043
Horizontal cup-to-disc ratio †	0.56 ± 0.08	0.54 ± 0.07	0.03 (0.01, 0.04)	0.031	0.070
Vertical cup-to-disc ratio †	0.52 ± 0.08	0.51 ± 0.06	0.01 (−0.01, 0.03)	0.315	0.443
Vertical disc diameter (μm) †	1337.18 ± 184.82	1302.82 ± 174.45	32.26 (8.00, 54.00)	0.010	0.033
Horizontal disc diameter (μm) †	1263.55 ± 189.31	1167.61 ± 169.36	94.29 (52.50, 138.50)	< 0.001	0.001
Neuroretinal rim Width (μm) †	292.88 ± 39.07	291.00 (277.00, 305.00)	7.00 (−1.00, 14.00)	0.094	0.163
Vertical cup diameter (μm) †	700.06 ± 179.70	666.55 ± 152.25	31.21 (6.50, 68.00)	0.064	0.118
Horizontal cup diameter (μm) †	722.33 ± 211.15	637.00 ± 155.33	78.50 (42.00, 121.00)	< 0.001	0.009
Disc tilt ratio	1.07 (1.02, 1.13)	1.13 (1.06, 1.19)	−0.06 (−0.08, −0.03)	< 0.001	0.006

Disc tilt ratio, the min-to-max disc diameter ratio; PPA, peripapillary atrophy; SE, Spherical Equivalent.

† *P*-value and Difference (95% CI) were calculated by *t*-test. Others were calculated by Wilcoxon signed rank test and Hodges-Lehmann estimator. FDR corrections (the Benjamini-Hochberg method) were done for multiple outcomes in anisometropic patients.

Regarding retinal vascular parameters, the average venous tortuosity was significantly lower in the lower-SE eyes compared to the higher-SE eyes (*P* = 0.023), while the total vessel length density was higher (*P* = 0.001). For fractal dimensions, the arterial D0 and D1 values were significantly higher in the lower-SE eyes (*P* = 0.016, *P* = 0.035, respectively). This indicates that the retinal arterial network in lower-SE eyes had a higher degree of spatial occupancy and more inhomogeneous distribution.. No statistically significant intergroup differences were found for the remaining parameters, including vessel area density, arteriolar-to-venular ratio (AVR), average branching angle, average branch asymmetry, average number of branches for arteries and veins, and venous fractal dimensions (all *P* > 0.05) (Table 4).

**TABLE 4:** Characteristics of the vasculature in myopic anisometropic patients.

Parameters	Low-SE eyes	High-SE eyes	Difference (95% CI)	*P*-value	Adjusted *P*-value
length_avg_a †	125.17 ± 25.93	120.06 ± 25.32	7.72 (−5.35, 17.77)	0.352	0.478
curvature_avg_a †	1.05 ± 0.03	1.04 ± 0.03	0.02 (−0.00, 0.03)	0.135	0.223
length_avg_v †	126.10 ± 24.63	120.06 ± 24.98	7.32 (−5.67, 16.60)	0.272	0.398
curvature_avg_v †	1.05 ± 0.03	1.03 ± 0.03	0.02 (0.00, 0.03)	0.023	0.057
vessel_length_density †	0.01 ± 0.00	0.01 ± 0.00	−0.00 (−0.00, −0.00)	0.001	0.011
vessel_density †	0.12 ± 0.02	0.12 ± 0.01	−0.00 (−0.01, 0.00)	0.065	0.118
AVR	0.72 (0.67, 0.74)	0.70 (0.67, 0.73)	0.01(−0.03, 0.04)	0.791	0.834
angle_avg_a †	75.75 ± 34.07	69.56 ± 35.12	8.19 (−9.17, 25.72)	0.464	0.607
asymmetry_avg_a †	39.54 ± 24.55	37.74 ± 22.75	2.96 (−10.47, 14.76)	0.749	0.817
brunch_avg_a †	2.23 ± 1.18	2.31 ± 1.40	−0.13 (−1.20, 0.92)	0.812	0.834
angle_avg_v †	73.66 ± 27.51	69.64 ± 32.42	1.18 (−12.75, 17.79)	0.645	0.790
asymmetry_avg_v	42.11 (25.23, 50.55)	40.17 (25.22, 51.72)	−3.16 (−13.59, 9.79)	0.572	0.725
brunch_avg_v †	2.26 ± 0.98	2.01 ± 0.80	0.33 (−0.33, 1.13)	0.254	0.385
D0_a †	1.12 ± 0.04	1.13 ± 0.04	−0.02 (−0.03, −0.00)	0.016	0.044
D1_a †	1.07 ± 0.04	1.08 ± 0.04	−0.02 (−0.03, −0.00)	0.035	0.073
D2_a †	1.05 ± 0.04	1.06 ± 0.04	−0.01 (−0.03, 0.00)	0.064	0.118
SL_a †	0.87 ± 0.13	0.88 ± 0.15	−0.01 (−0.07, 0.06)	0.743	0.817
D0_v †	1.10 ± 0.04	1.10 ± 0.04	0.00 (−0.02, 0.01)	0.866	0.866
D1_v †	1.05 ± 0.04	1.05 ± 0.04	−0.00 (−0.02, 0.02)	0.753	0.817
D2_v †	1.03 ± 0.04	1.03 ± 0.04	−0.00 (−0.02, 0.02)	0.730	0.817
SL_v †	0.88 ± 0.11	0.85 ± 0.17	0.03 (−0.03, 0.08)	0.239	0.378

AVR, Artery to vein ratio; angle_avg, the average branch angle; asymmetry_avg:the average branch asymmetry; brunch_avg, the average number of branches; D0, Capacity dimension; D1, Entropy dimension; D2, Correlation dimension; SL, Singularity Length; with the suffix “_a” for artery-related metrics and “_v” for vein-related metrics; SE, Spherical Equivalent. † *P*-value and Difference (95% CI) were calculated by *t* test. Others were calculated by Wilcoxon signed rank test and Hodges-Lehmann estimator. FDR corrections (the Benjamini-Hochberg method) were done for multiple outcomes in anisometropic patients.

In summary, significant interocular differences existed between anisometropic eyes in FT distribution, PPA extent, optic disc morphology, arterial network complexity, and venous tortuosity. However, differences in most other retinal vascular structural parameters did not reach statistical significance.

### 3.3 Correlation analysis between the degree of anisometropia and interocular differences in fundus parameters

**FIGURE 4:**
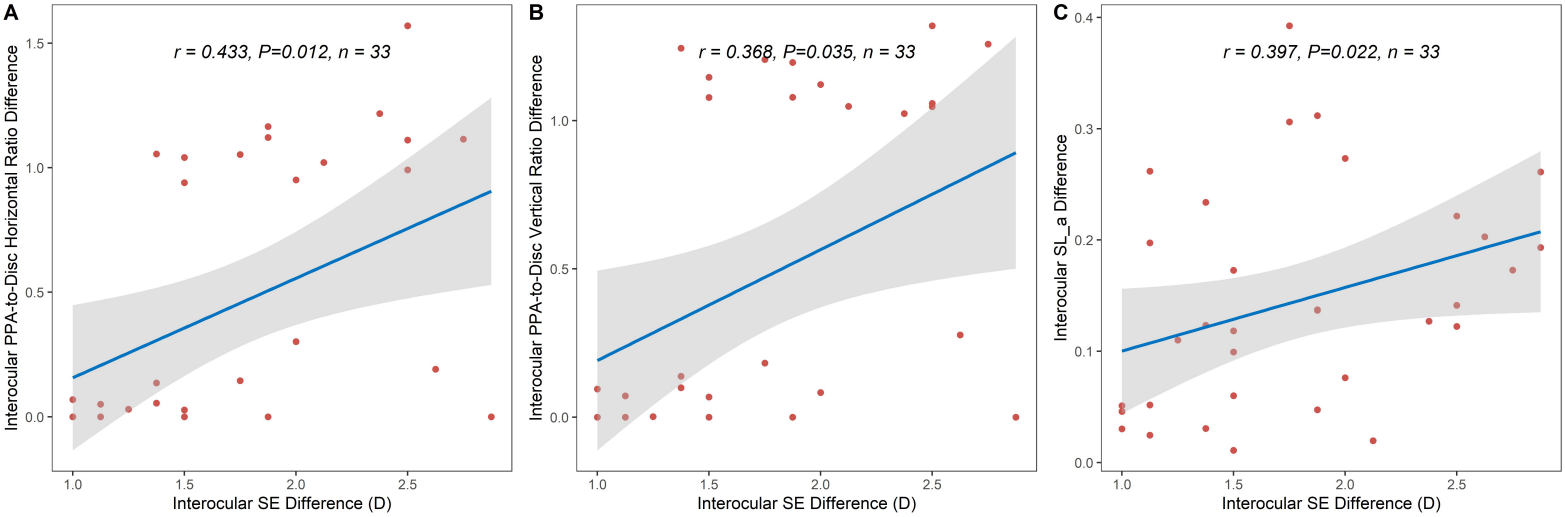
Scatter plots showing correlations between interocular differences in spherical equivalent (SE) and retinal parameters. **(A)** Horizontal diameter ratio of the PPA to the optic disc. **(B)** Vertical diameter ratio of the PPA to the optic disc. **(C)**:SL_a; Data points represent absolute interocular differences for each parameter, only correlations that reached statistical significance are presented; D, Diopter.

We investigated the association between interocular differences in various fundus indicators and the degree of anisometropia (interocular SE difference). The results showed that the interocular differences in both the horizontal and vertical diameter ratios of PPA to the optic disc were positively correlated with the degree of anisometropia (*r* = 0.433, *P* = 0.012; *r* = 0.368, *P* = 0.035, respectively). This indicates that a greater degree of anisometropia was associated with a larger interocular difference in the PPA-to-optic disc diameter ratios. The interocular difference in arterial Singularity Length (SL_a) also showed a positive correlation with the degree of anisometropia (*r* = 0.397, *P* = 0.022), suggesting that greater anisometropia was linked to a larger interocular difference in the local structural irregularity of arteries. No significant correlations were found between the degree of anisometropia and the interocular differences in other fundus parameters (all *P* > 0.05) (Figure 4).

## 4 Discussion

Anisometropia is a significant factor affecting visual development in children. If its degree is substantial and remains uncorrected during childhood, it can impair the development of binocular visual function and lead to amblyopia (27). Furthermore, anisometropia is associated with various visual problems, including aniseikonia, spectacle intolerance, visual fatigue, reduced stereopsis, and sub-normal binocular function (28). Current research investigating the structural basis of anisometropia has primarily focused on interocular differences in axial length (1, 29). Systematic studies on interocular differences in fundus morphology among patients with different types of anisometropia remain relatively limited. Therefore, this study employed AI technology to conduct a quantitative analysis of CFP images from both eyes of children with mild to moderate anisometropia. We aimed to explore the interocular differences in fundus parameters in this patient group and further analyze their correlation with the degree of anisometropia.

Regarding the degree of FT, this study found that eyes with lower SE values exhibited higher FT density and a larger affected area. FT is defined as the clear visibility of larger choroidal vessels in the posterior pole of the retina. It serves as a straightforward method for directly visualizing the choroidal vascular structure (30–33). Its formation mechanism is primarily related to choroidal thinning caused by axial elongation. In a normal eye, the retinal pigment epithelium and melanocytes within the choroid effectively obscure the underlying vessels. As the axial length increases and choroidal thickness decreases, this pigmentary obscuring effect weakens. This enhances the visibility of choroidal vessels, thereby making the tessellation sign more apparent (34). Previous studies support the correlation between FT and the degree of myopia. For instance, Li et al. applied AI technology to quantitatively assess FT density in adolescents and found a significant negative correlation with SE (10). Similarly, Shao et al. (12) demonstrated a significant negative correlation between FT density and SE in myopic individuals, while no such association was observed in hyperopic or emmetropic populations (12). The significant interocular difference in FT degree observed in our anisometropic patients suggests that this indicator may serve as an early morphological marker for anisometropia.

Abnormal vascular tortuosity is often associated with vascular dysfunction and breakdown of the blood-retinal barrier (35, 36). This study found that the average venous tortuosity was significantly lower in lower-SE eyes compared to higher-SE eyes. This phenomenon may result from mechanical stretching caused by axial elongation of the eyeball (37, 38). Regarding vascular network complexity, lower-SE eyes showed higher total vessel length density and significantly higher arterial fractal dimensions (D0, D1) relative to higher-SE eyes. This suggests that the retinal arterial network in lower-SE eyes has a higher degree of spatial occupancy and a more uneven distribution. However, no significant interocular differences were found in parameters such as vessel branching angles and the AVR. These results indicate that in patients with mild to moderate anisometropia, interocular differences in retinal vascular morphology exhibit certain characteristic features, but these changes are not typical or consistent across all parameters. Findings from previous studies on retinal vascular parameters in myopic patients are also somewhat contradictory. Zhao et al. found no significant association between the retinal arteriolar-to-venular diameter ratio and axial length in myopic children (5). In contrast, a study by Yii et al. (39) based on a large cohort of healthy adults found that as the negative refractive error increased, the degree of central retinal venular equivalent dilation was significantly greater than that of arterioles, and the fractal dimension decreased significantly, particularly in high myopia (39). The discrepancy between that study and our findings may be attributed to differences in the study populations. The study by Yii et al. was based on an adult cohort and included high myopia, whereas our study focused on school-age children and excluded high myopia. We speculate that in the early stage of anisometropia, the lower-SE eye may increase the density and complexity of the superficial capillary network to compensate for potential relative choroidal-retinal blood flow insufficiency caused by axial elongation. This compensatory mechanism might aim to maintain oxygen and nutrient supply to the inner retinal layers (e.g., nerve fiber layer, ganglion cell layer, inner plexiform layer), thereby attempting to preserve visual function under the condition of anisometropia (40, 41). In summary, the sensitivity and stability of retinal vascular morphology as an indicator for predicting or monitoring anisometropia require further validation.

In our study of optic disc morphology in anisometropic patients, we found that distinct interocular differences were already present at an early stage. Specifically, lower-SE eyes exhibited a larger area ratio of PPA to the optic disc, increased PPA width and height, smaller optic disc diameters, a larger optic disc ovality index, and a slightly lower horizontal cup-to-disc ratio. The optic disc ovality index is defined as the ratio of the longest to the shortest diameter of the optic disc. An ovality index > 1.3 is typically defined as optic disc tilt (42). A study by Samarawickrama et al., analyzing stereoscopic fundus photographs of 1227 adolescents, found that the degree of optic disc tilt was closely associated with more negative spherical power, longer axial length, and more negative cylindrical power. Morphologically, that study further showed that eyes with tilted discs had significantly smaller horizontal optic disc and cup diameters compared to eyes without tilt, leading to significantly lower horizontal and vertical cup-to-disc ratios (43). These findings are consistent with our conclusions. Notably, in our correlation analysis, the interocular difference in the PPA-to-optic disc diameter ratio significantly increased with a greater degree of anisometropia. This suggests that this parameter could serve as an objective, quantifiable morphological marker reflecting the severity and potentially monitoring the progression of anisometropia. It may have potential value as an early warning indicator for the identification and monitoring of anisometropia progression. Previous research has preliminarily explored the association between myopic anisometropia and PPA. Qiao et al. (2023) found that in patients with myopic anisometropia, the γ-PPA area was significantly larger in the more myopic eye (*P* = 0.002). Furthermore, the interocular difference in γ-PPA area showed a significant positive correlation with the interocular difference in axial length (*r* = 0.361, *P* = 0.015). Although the β-PPA area was also larger in the more myopic eye (*P* = 0.045), its interocular difference did not correlate significantly with the axial length difference (*P* > 0.05) (44). Our study extended the sample to include hyperopic and mixed anisometropia and similarly observed a correlation between PPA extent and the degree of anisometropia. This suggests that such structural changes might be a common feature across different types of anisometropia. The precise mechanisms underlying the interocular differences in PPA morphology in anisometropia are not yet fully understood. During the school-age period, the posterior segment of the eye undergoes predominantly axial elongation, particularly in the equatorial region, with the ocular shape gradually transitioning from spherical to ellipsoidal (45). Existing theories propose that PPA enlargement may be related to mechanical stretching from axial elongation, particularly the posterior slippage of the sclera due to mismatched growth between the retina and sclera (46). Additionally, PPA formation may also be associated with changes in choroidal thickness. A study by Chen et al. on young myopic patients found that the prevalence of PPA increased with longer axial length, and an increase in PPA area was accompanied by a decrease in choroidal thickness in both the macular and peripapillary regions (47). Based on these findings, we speculate that the developmental process of axial elongation may already be progressing at divergent rates between the two eyes in patients with mild to moderate anisometropia. Future studies are warranted to further validate the above mechanisms in anisometropic populations and to analyze the dynamic relationship between PPA, ocular development, and anisometropia progression through long-term follow-up. Moreover, axial length should be incorporated as a key parameter in future research to further verify the reliability of the interocular PPA difference as a fundus biomarker for monitoring anisometropia progression.

The innovation of this study lies in its use of AI-based quantitative analysis to systematically reveal characteristic changes in interocular fundus structural parameters among children with mild to moderate anisometropia. It should be emphasized that the purpose of this work is not to replace conventional vision tests or cycloplegic refraction in school screening programs. Rather, we explored whether fundus morphological differences detected by AI could serve as an objective imaging adjunct to existing subjective approaches. Routine refraction relies heavily on children’s cooperation, examiner experience, and the implementation of cycloplegia, whereas color fundus photography is non-invasive, rapid, and places lower compliance demands on young children. Therefore, if the quantitative relationships between certain fundus parameters (such as the peripapillary atrophy-to-optic disc diameter ratio) and the degree of anisometropia can be firmly established, AI-based fundus image analysis may offer an objective imaging reference for early identification and longitudinal monitoring of anisometropia, thus complementing traditional screening procedures. Moreover, from a methodological perspective, the paired-eye design employed in this study constitutes an important strength in itself: each participant’s fellow eye serves as the ideal control, such that all intrinsic, individual-level confounders that do not vary between eyes—including age, sex, ethnicity, and other demographic characteristics—are naturally and fully eliminated when interocular differences are calculated. This finding primarily benefits from the wide accessibility of CFP technology and the quantitative analytical capabilities of AI image processing. We have preliminarily identified several fundus indicators that show significant interocular differences in anisometropia. This lays the groundwork for subsequent exploration of imaging biomarkers for the early identification of anisometropia and provides new perspectives for monitoring its progression. However, this study has several limitations. First, the relatively limited sample size not only constrains statistical power but also precludes meaningful subgroup analyses by refractive subtype, which may partly explain why some findings did not reach statistical significance. The paired-eye design, while controlling inter-individual variation, may also lead to an underestimation of certain associations. Second, the cross-sectional design limits causal inference; moreover, due to the retrospective nature of the study, axial length data were not available for all participants at the same visit, precluding the inclusion of this core parameter that links anisometropia to fundus structural changes. Third, with regard to methodological considerations, we did not adjust for interocular differences in optic disc size, which may have confounded certain morphological outcomes; the 45-degree optic-disc-centered fundus images may restrict comprehensive assessment of the peripheral vasculature, and wider-field imaging is desirable in future work; furthermore, although the AI-based approach overcomes the subjective bias inherent in manual measurement, it may still yield false-positive and false-negative results, necessitating further research on its safe, effective, convenient, and cost-effective integration into clinical practice. Future large-scale, prospective cohort studies that enroll children without anisometropia at baseline for longitudinal follow-up, and that incorporate interocular axial length differences into the analysis, are warranted to validate these findings.

## 5 Conclusion

This study employed AI technology to conduct a quantitative analysis of color fundus photographs from both eyes of children with mild to moderate anisometropia. It systematically revealed significant interocular differences in fundus structural parameters, including fundus tessellation, optic disc morphology, and retinal vascular characteristics. Notably, the correlation analysis demonstrated a significant linear association between the degree of anisometropia and the magnitude of the interocular difference in the PPA-to-optic disc diameter ratio. In summary, AI-based quantitative analysis of fundus images offers a promising objective imaging tool for the early identification and follow-up monitoring of anisometropia; however, given the limitations of the current study, particularly its sample size, further validation through larger-scale longitudinal studies is warranted.

## Data Availability

The raw data supporting the conclusions of this article will be made available by the authors, without undue reservation.
